# Isotretinoin in Thick-Skin Rhinoplasty: A Review

**DOI:** 10.1055/s-0044-1801319

**Published:** 2025-04-22

**Authors:** Rafael Pessoa Porpino Dias, Jose Luiz Teixeira Rodrigues, Maíra Said Dias Jabour, Raíssa de Oliveira e Albuquerque, Henrique de Almeida Friedrich, Michele Sander Westphalen, Carlos Alberto Caropreso

**Affiliations:** 1 Department of Ophthalmology, Otorhinolaryngology, Head and Neck Surgery, Universidade de São Paulo (USP), São Paulo, SP, Brazil

**Keywords:** thick-skin rhinoplasty, isotretinoin, rhinoplasty, thick skin

## Abstract

**Introduction** Thick-skinned patients are prevalent in the general population worldwide and represent a challenge for the rhinoplasty surgeon. The use of oral isotretinoin before and/or after surgery is a possible adjuvant treatment that can facilitate intraoperative management and improve results in the postoperative stages; however, there are still questions about its effects in the long-term.

**Objective** To evaluate the advantages and best practices for orally administrated isotretinoin in thick-skin rhinoplasty based on the current literature.

**Methods** An advanced search was conducted until August 31st, 2023, on the PubMed, Cochrane Library, ClinicalTrials.gov, Embase, and LILACS databases with the keywords *rhinoplasty* and *isotretinoin* . Sixteen articles were reviewed and 4 met the inclusion criteria, with a total of 371 patients. Meta-analysis of the data collected was not possible due to heterogeneity among papers.

**Conclusion** Isotretinoin can be a safe drug, and its use in rhinoplasty varies widely, although all studies reported a low-dose oral regime for up to 6 months. The medication use is well-established in thick-skin rhinoplasty. Small doses after the procedure can improve patient satisfaction and the quality and thickness of the skin in the first 6 months of postsurgery. After 6 months, studies with a control group did not show a significant difference in patient satisfaction rates. A strong framework and specific surgical maneuvers may be more important than isotretinoin for better outcomes in thick-skin rhinoplasty.

## Introduction

Rhinoplasty is one of the most common procedures in cosmetic surgery; more than 200,000 were performed in the United States in 2018, making it the third most common plastic surgery in that country. 1 2 Routinely, patients who seek this procedure are young. Some of them suffer from oily and acne-prone skin. 3 This condition is commonly associated with thick skin and soft-tissue envelope (SSTE), which can increase complication rates. 4 5 6 7 8

To reduce the risks of poor outcomes, it is essential for surgeons to evaluate the thickness of SSTE in each patient and plan adequate preparation for them individually. Clinically, skin width can be divided into three categories: thick, moderate, and thin; so, different operative techniques and postoperative management can be applied. 3 4 5 6 To ensure good aesthetic results in rhinoplasty, proper nasal skeleton contour, and SSTE redraping are necessary; unfortunately, that is a challenge in thick-skinned patients. 9 10 11 In this group, severe nasal edema, loss of tip definition, fibrosis, and pollybeak deformity are possible complications associated with “dead space” phenomenon, which is when adequate redraping is not possible. Moreover, when present, these problems are difficult to treat. 12

To properly address thick SSTE in rhinoplasty, there are various adjuvant medical options, including those that reduce sebaceous gland hyperplasia aiming to obtain a better-defined nose tip. 3 4 5 13 One of the most famous and effective medications in this context is oral isotretinoin, a retinoid that leads to reduction in sebum production and comedogenic activity. 3 14 Despite the considerable list of side effects as teratogenicity, xerosis, and granulation tissue, its use in rhinoplasty has demonstrated to be safe and well tolerated. 15 16 In addition, low-dose regimens from 0.25 to 0.5 mg/kg daily have demonstrated to be as efficient as the standard dosing regime and may increase tolerance and patient adherence. 17 Although there are a great number of papers reporting the use of oral isotretinoin in rhinoplasty, there are no review articles to answer the question: is oral isotretinoin beneficial in thick-skin rhinoplasty in the long term?

## Review of the Literature

A review was conducted until August 31st, 2023, using the following keywords: *rhinoplasty* AND *isotretinoin* in the PubMed, Cochrane Library, ClinicalTrials.gov, Embase, and LILACS databases. All publications in English that related the use of isotretinoin to rhinoplasty were included. The exclusion criteria were phase-3 trials with no published outcomes, reviews, and expert opinion articles, as well as studies on other topics. Since the present study is a retrospective analysis and literature review, research ethics authorization was not required.

**Fig. 1:**
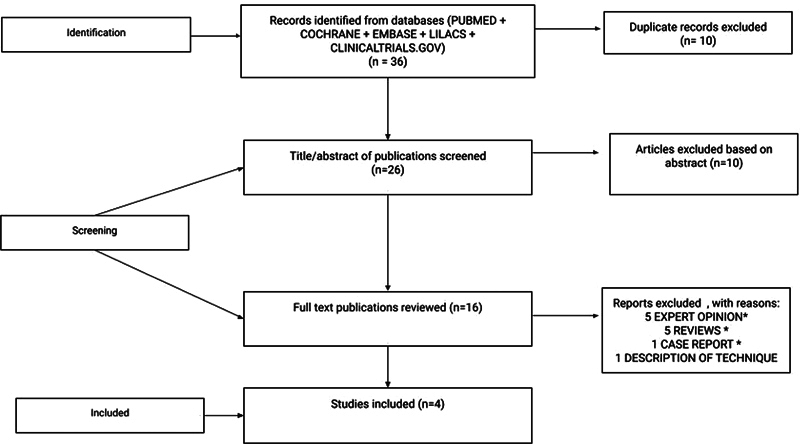
Preferred Reporting Items for Systematic reviews and Meta-Analyses (PRISMA) flow diagram. **Abbreviation:** SLR, systematic literature review.

Every article's level of evidence was evaluated using the Oxford Center for Evidence-Based Medicine (CEBM) criteria. Using the terms *rhinoplasty* AND *isotretinoin* , 36 articles were found in the first search conducted in the previously mentioned databases. After 10 duplicate articles were eliminated, 26 articles remained. Eligible articles were published in English and were clinical trials, case series, or cross-section studies. Non-related review articles, specialist opinions, animal model articles, and manuscripts were excluded. Using the specific eligibility and exclusion criteria mentioned, four articles were included in a database, two of which were interventional and two cross-sectional studies, with a total of 371 patients. The publication period covered was from 2005 until 2022. The current study was performed as per the Preferred Reporting Items for Systematic Reviews and Meta-analysis (PRISMA), as shown in Fig. 1 .

## Discussion

Although there were numerous articles about oral isotretinoin use in rhinoplasty, only two were randomized controlled studies; both were selected but neither had a unified scale measure for objective end-point evaluation. The other two selected articles have a low quality of evidence. All four of them will be discussed in chronological sequence; afterwards, some points concerning thick-skin rhinoplasty will be addressed based on other important published articles not included in this review.

In 2005, Allen and Rhee 18 published a retrospective analysis of 3 cases of patients who developed nasal tip deformities after oral isotretinoin use later to rhinoplasty. The surgical techniques used were not properly described, but no specific approach to treat nasal skin was mentioned. Among the complications, they 18 described nasal tip deformity, alar collapse, skin thinning, prominence of cartilage grafts, and nasal tip edema. However, the small number of patients, the retrospective analysis, and the lack of a comparison group are some troublesome limitations. In addition, one of the presented cases was a patient who had undergone two previous rhinoplasties. Another one was an individual with a congenital deformity of the nose, who had undergone a rhinoplasty with a composite graft to reconstruct the soft-tissue triangle. It is possible that the nasal tip deformities in those two cases would be present despite the use of isotretinoin.

Allen and Rhee 18 described that the use of isotretinoin after rhinoplasty may predispose individuals to deformities by thinning the nasal tip skin through its interactions with collagen in the dermis, leading to increased contracture, even though, the use of isotretinoin may just have accelerated or accentuated the contracture phenomenon of postoperative healing.

A summary of the findings and study design can be seen in Table 1 , along with the other selected papers.

**Table 1:** Summary of the included articles

Article	Study design	Level of evidence	Sample size	Isotretinoin			Rhinoplasty outcome
				Dose	Initiation	Duration	
Complications associated with isotretinoin use after rhinoplasty (Allen and Rhee, 2005 18 )	Case series	4	3	undisclosed	postoperative	6 months	Skin thinning, nasal tip asymmetry, alar collapse, and nasal tip bossae
Isotretinoin use in thick-skinned rhinoplasty patients (Cobo and Vitery, 2016 19 )	Case series	4	17	0.25 to 1 mg/kg/day*	postoperative	4 to 6 months	Significant improvement in the appearance and the texture of the skin. More defined nasal tip than presurgical images
Oral isotretinoin in the treatment of postoperative edema in thick-skinned rhinoplasty: a randomized placebo-controlled clinical trial (Sazgar et al., 2019 21 )	Double- blinded placebo- controlled clinical trial.	2	48	0.5 mg/kg every other day for 1 month; and then 0.5 mg/kg/day for 2 months	postoperative	3 months	Significant improvement in patient satisfaction and specialist-reported aesthetic outcome at three and six months after surgery in the intervention group ( *p* < 0.05); Twelve months after surgery, there was no statistically significant difference between the control and the intervention groups
Analysis of the effects of isotretinoin on rhinoplasty patients (Yahyavi et al., 2020 3 )	Randomized control trial	3	303	0.3 mg/kg/day for 2 weeks before surgery until 2 months after surgery	pre operative	2.5 months	Significant improvement in patient satisfaction ( *p* < 0.01) and facial skin oiliness ( *p* < 0.05) at three and six months after surgery in the intervention group

**Notes:** A total of 4 studies and 371 patients were included. *Two cases required doses of 1 mg/kg/day, while 15 patients received doses ranging from 0.25 mg/kg/day to 0.5 mg/kg/day.

In 2016, Cobo and Vitery 19 published a retrospective study with 17 thick-skinned patients who had rhinoplasties done and received oral isotretinoin during the first months after surgery. All of the patients were operated on by the senior author using an open approach and structural philosophy. The underlying bony and cartilaginous structure was reinforced with grafts and sutures. All of them had thick and acne-prone skin and had wide bulbous undefined nasal tips with fragile cartilaginous structures. They were monitored and received a dose ranging from 0.25 mg/kg to 0.5 mg/kg for 4 to 6 months. Pictures were taken at 6 and 12 months, as well as every year after surgery.

Patients had a significant improvement in the appearance of nasal and facial skin. None of them received additional treatments (steroid injections), and none of them had scarring or abnormal scars. 19 It was concluded that oral isotretinoin has a positive impact, especially in mestizo/Hispanic, Middle Eastern, and Asian patients, in which the underlying fibroadipose tissue plays an important role. In addition, it can be useful for adolescents and young adults, patients with commonly acne-prone skin.

After rhinoplasty, acne can be exacerbated by poor cleansing routines and prolonged use of nasal tapes. Isotretinoin could be an option for improving nasal tip definition and also controlling acne exacerbations without exposing patients to unpredictable procedures, such as partial superficial muscular aponeurotic system (SMAS) resection or resection of the subcutaneous fibro adipose tissue of the tip-supra tip area. 19 Those findings are corroborated by other studies 5 17 20 that have recommended dosages from 0.25 to 0.5 mg/kg daily.

In 2019, Sazgar et al. 21 published a double-blind placebo-controlled trial comparing preoperative and postoperative photography in two patient groups: patients treated with oral isotretinoin and with a placebo. Surgeries were performed by the senior author (AAS) using an open approach. Patients underwent common surgical techniques such as bony and cartilaginous hump excision, auto spreader flap, tongue in groove, nose tip sutures, and cephalic trim of the cephalic portion of the lateral crus of the lower lateral cartilage. Taping, skin sutures, and nasal splints were removed on the 6th day following the surgery. No other external care was prescribed afterward.

Photographs were compared at 3, 6, and 12 months by a facial plastic expert blinded to the patient groups. Patients reported their cosmetic satisfaction also at 3, 6, and 12 months after surgery. They started with 0.5 mg/kg of isotretinoin every other day for 1 month and then continued with 0.5 mg/kg per day for two additional months.

They 21 found that patient satisfaction and cosmetic surgical outcome in the isotretinoin group at 3 and 6 months after surgery were both significantly higher than in the placebo group. However, 12 months after surgery, there was no statistically significant difference between the groups.

Regarding isotretinoin treatment side effects, two patients complained of nasal dryness, and one patient had bloody discharge. No patient had any significant changes in laboratory findings after the medication. They concluded isotretinoin can accelerate the improvement in cosmetic results and patient satisfaction during the first 6 months after surgery. It can also decrease patient anxiety, providing better interaction between the surgeon and patient and a faster return to social relations. However, the long-term surgical result seems more affected by the creation of a strong cartilaginous framework.

Sazgar et al. 21 affirm that one of the main concerns about the perioperative use of isotretinoin is its possible interference with skin healing. Plastic surgeons commonly believe that oral isotretinoin should be stopped at least 6 to 12 months before elective surgery to avoid possible wound healing impairment, but this study points out that this time could be abbreviated. Moreover, it stresses the need for future studies to help determine skin thickness before and after intervention by ultrasonography or computed tomography scan while also evaluating patient satisfaction using validated questionnaires.

Finally, in 2020, Yahyavi et al. 3 analyzed the effects of oral isotretinoin on 303 rhinoplasty patients. Only autogenous cartilage grafts were used during the surgery, and all patients had strut grafts placed. Alar reduction was performed on 98% of patients. No specific surgical maneuvers for thick rhinoplasty were reported and all of the surgical operations were carried out by one surgeon (SY), under general anesthesia with an endonasal approach. The dosage used was nearly 0.3 mg/kg daily (equivalent to a 20-mg capsule of isotretinoin). They started to use isotretinoin 2 weeks before surgery, to control facial skin oiliness during the early postoperative period and discontinued it after 2 months.

Other studies 5 started the medication 3 to 4 weeks after surgery and continued for 4 to 5 months, but the authors believe that when it is used in the immediate postoperative period, it offers more benefits.

Since isotretinoin administration could cause granulation and disturbance of wound recovery, this drug was avoided in the perioperative period of facial surgery for many years. The recommendation was to stop its administration from 6 to 24 months before surgery. 22 23 24 25 26

Nevertheless, Ungarelli et al. 16 studied 47 articles and concluded that isotretinoin does not promote skin healing complications and considered that discontinuation of the medication for 30 to 35 days before surgery would be enough.

Of the 149 patients in the experimental group in the study by Yahyavi et al., 3 none showed cartilage deformities, keloid tissue, delay in the repair process of healing, or abnormal scars. The authors 3 used a Likert scale for nose appearance, and an arbitrary scale from 1 to 5 for skin oiliness and severity of facial acne. All of the patients had a significant improvement on those scales.

It was demonstrated that facial skin oiliness was lower in the first- and third-months following rhinoplasty when patients used isotretinoin, compared with a control group; still, this result was not consistent 6 and 12 months after the surgery. In previous studies, 27 it has been already documented that acne increased by 27% in the first month in patients who underwent rhinoplasty, compared with those who had only functional nasal surgery done, and Yahyavi et al. 3 pointed out the benefit of isotretinoin for this problem.

When comparing postoperative satisfaction, the experimental group had significantly higher rates than the control group in the first and third months after surgery ( *p* < 0.01). Despite that, one year after surgery, the satisfaction of both groups was similar. 3

The papers that met inclusion criteria unfortunately did not elucidate properly the long-term effect of isotretinoin in the skin or specific surgical steps when the drug is used. However, in 2017, Guyuron and Lee 7 had already postulated an algorithm for the management of thick-skin rhinoplasty based on their experience. They 7 brought a series of steps to follow evaluation of the skin, medication treatment, domal fat pad removal, strong nasal framework, dead space elimination, redundant skin removal, and postoperative care. They recommend adequate skin treatments such as proper diet (avoid hyperglycemic products, dairy, saturated and trans fats), topical isotretinoin, and oral isotretinoin, under a dermatologist's care. Patients with thick skin should have a thinning of the SMAS done, controlled removal of the subcutaneous fat between the domes, and a firm underlying nasal tip frame (with graft reinforcement). Dead space elimination can be accomplished during surgery by a gently tied suture of the deep subcutaneous tissue in the supra tip area and the anterior septal angle; and later by taping the supra tip area after work hours and the weekends for 30 to 60 days. 7 This last measure has already been proven effective in controlling postoperative edema in thick-skin rhinoplasty in a randomized controlled trial. 28

In 2021, Patrocinio et al. 29 published1 their perceptions of rhinoplasty in African descendants, a population known for thick SSTE, and published the advances in this area. They reinforced the improvement in patients' satisfaction and nose appearance in the first months after surgery when oral isotretinoin was used, even though no significant change was present in one year. Additionally, they 29 advocated in favor of using an open approach, using costal cartilage for grafts, and accessing the inferior lateral cartilage through supraperichondral dissection. Controlled debulking, preservation of the Pitanguy ligament, septal extension graft use, and nasal tip graft (shield and cap grafts) placement are mentioned as possible surgical techniques for achieving better results in patients with thick skin too. The preserved deep Pitanguy ligament on the flap helps to increase supra tip break and prevent pollybeak deformity when it is reinserted between vestibule mucosa and the medial crura using 4-0 polydioxanone sutures. 30 Taping at night and subcutaneous injections of triamcinolone (0.2–0.4 ml of 10 mg/ml) after surgery are presented as tools to help decrease supra tip edema. 31

Later, using ultrasonography and elastography, Yigit et al. 20 published a controlled trial evaluating the impact of different systemic isotretinoin doses (0.25 mg/kg/day and 0.5 mg/kg/day) on nasal skin thickness and elasticity in acne vulgaris patients. After 2 and 4 months of treatment, dermis and subcutaneous soft-tissue width decreased at each nasal anatomical landmark, regardless of the dosage used. 20 Since skin elasticity, thickness, and quality are among the biggest challenges for optimal rhinoplasty results, especially on nasal tip definition, it is useful to know that 4 months of low-dose isotretinoin treatment can be as effective as higher doses. 20 32 33 There is additional relevance once possible adverse effects of isotretinoin are dose-dependent. 20

## Conclusion

Isotretinoin use in rhinoplasty varies widely in dose, initiation time, and duration, but most of the studies reported a low dose for up to 6 months. 3 19 20 21 Even though the medication has considerable adverse effects, current papers 15 16 17 show its safety, particularly when in a low-dose regime.

We could not conduct a meta-analysis of the data collected due to heterogeneity among papers; still, the medication use is well established in thick-skin rhinoplasty. It has been shown that small doses used after the procedure can improve patient satisfaction, and the quality and thickness of the skin in the first 6 months postsurgery. However, after 6 months, most studies with a control group did not show a significant difference in patient satisfaction rates. A strong framework and specific surgical maneuvers may be more important than isotretinoin use. 3 4 5 16 18 20 33

Oral isotretinoin is a possible adjuvant treatment for thick-skin rhinoplasty, but there are still insufficient high-quality papers to determine its optimal dose, duration, and long-term benefits.
